# Atom Transfer Radical Polymerization in the Solid‐State

**DOI:** 10.1002/anie.202005021

**Published:** 2020-06-08

**Authors:** Hong Y. Cho, Christopher W. Bielawski

**Affiliations:** ^1^ Center for Multidimensional Carbon Materials (CMCM) Institute for Basic Science (IBS) Ulsan 44919 Republic of Korea; ^2^ Department of Chemistry Ulsan National Institute of Science and Technology (UNIST) Ulsan 44919 Republic of Korea; ^3^ Department of Energy Engineering Ulsan National Institute of Science and Technology (UNIST) Ulsan 44919 Republic of Korea

**Keywords:** ball milling, controlled radical polymerization, modeling, polymer decomposition, solid-state chemistry

## Abstract

Poly(2‐vinylnaphthalene) was synthesized in the solid‐state by ball milling a mixture of the corresponding monomer, a Cu‐based catalyst, and an activated haloalkane as the polymerization initiator. Various reaction conditions, including milling time, milling frequency and added reductant to accelerate the polymerization were optimized. Monomer conversion and the evolution of polymer molecular weight were monitored over time using ^1^H NMR spectroscopy and size exclusion chromatography, respectively, and linear correlations were observed. While the polymer molecular weight was effectively tuned by changing the initial monomer‐to‐initiator ratio, the experimentally measured values were found to be lower than their theoretical values. The difference was attributed to premature mechanical decomposition and modeled to accurately account for the decrement. Random copolymers of two monomers with orthogonal solubilities, sodium styrene sulfonate and 2‐vinylnaphthalene, were also synthesized in the solid‐state. Inspection of the data revealed that the solid‐state polymerization reaction was controlled, followed a mechanism similar to that described for solution‐state atom transfer radical polymerizations, and may be used to prepare polymers that are inaccessible via solution‐state methods.

## Graphical abstract

**Solid‐state ATRP**: An atom transfer radical polymerization (ATRP) was performed in the solid‐state. The reaction is promoted by ball milling, found to proceed in a controlled fashion and facilitates access to copolymers that are challenging to prepare via solution‐state methods.

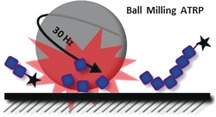

## Introduction

**Scheme 1:**
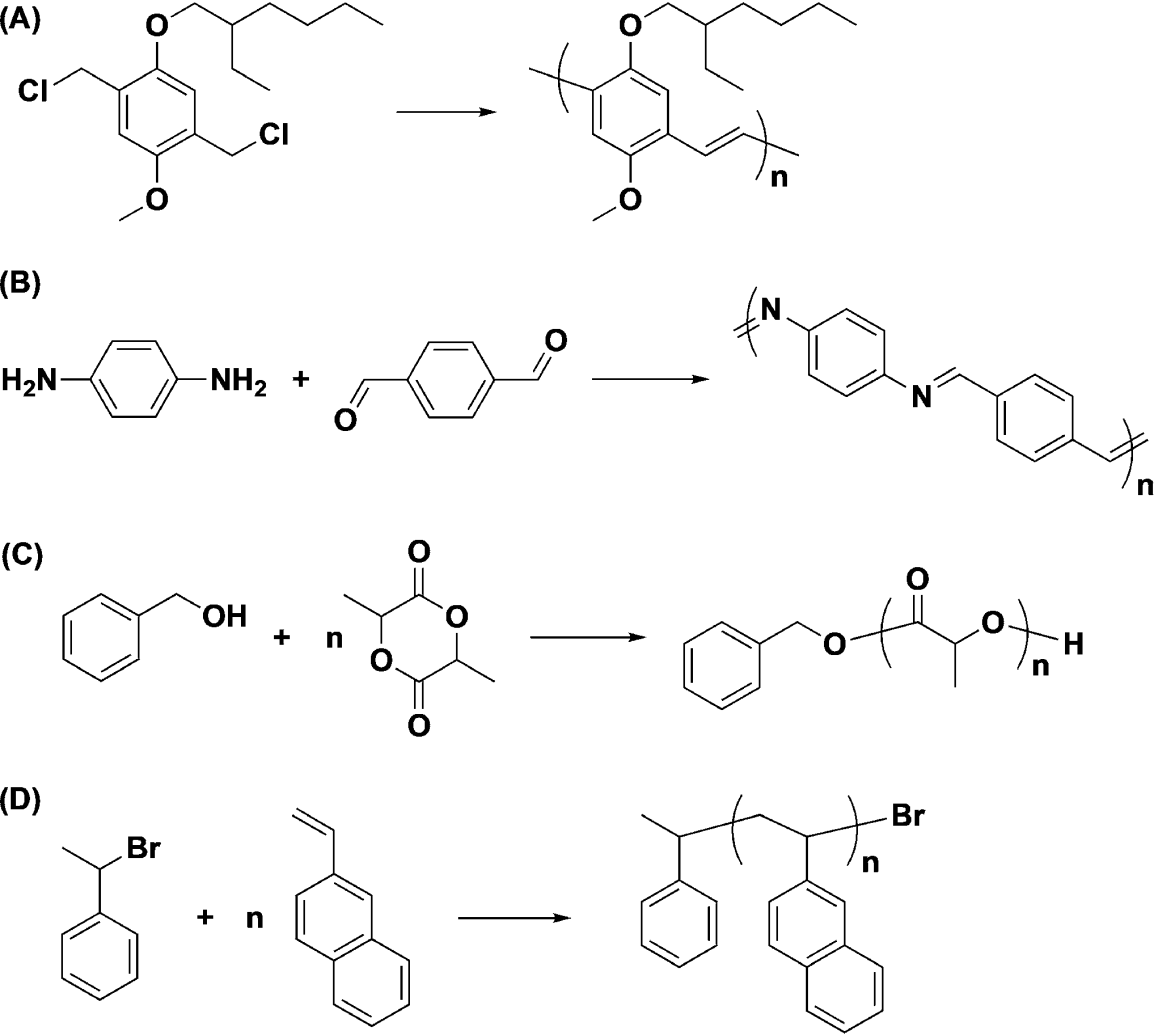
Examples of solid‐state (A) Gilch, (B) polycondensation, (C) ring‐opening polymerization, and (D) atom transfer radical polymerization reactions that are promoted by ball milling.

Ball milling (BM) processes have garnered attention because they can provide efficient and environmentally‐friendly alternatives to solution‐based reactions.^1^ The efficacy has been attributed to the high forces generated under BM conditions which effectively facilitate a broad range of chemistry, including organic^2^ and organometallic transformations,^3^ crystallization phenomena,^4^ and other productive chemical processes.^5^, ^6^ The majority of BM reports entail small molecule reactions and, by comparison, synthetic polymerization reactions have been relatively unexplored.^7^ An early example was disclosed by Swager, who demonstrated that BM facilitates the Gilch polymerization of 2‐methoxy‐5‐2′‐ethylhexyloxy phenylene vinylene in the solid‐state (Scheme 1 A).7a The methodology afforded the expected polymeric products in relatively high molecular weight (MW) (≈40 kDa) and under mildly basic conditions when compared to analogous reactions that were performed in the solution‐state. Borchardt subsequently described solvent‐free methods based on BM for condensing diamines and dialdehydes to afford poly(azomethine)s (Scheme 1 B). The solid‐state methodology obviated the need for high reaction temperatures and toxic solvents (e.g., hexamethylphosphoramide) commonly utilized in solution‐based processes for accessing the same polymeric products.7b Likewise, solid‐state polycondensations of dibromoarenes and dihalophenylboronic acid were found to proceed over shorter periods of time (0.5 h) when compared to analogous reactions performed in the solution‐state (12 to 24 h) and afforded a range of different architectures, including linear and hyperbranched poly(phenylene)s, in comparable yield.7c

The aforementioned reports demonstrated that the advantages intrinsic to BM processes may be used to drive step‐growth polymerizations and build upon analogous stoichiometric reactions that are promoted under similar conditions. Since BM has also been shown to promote various types of catalyzed transformations (e.g., olefin metathesis, coupling reactions, click chemistry, etc.),^8^ analogous methodology can be envisioned to enable chain‐growth polymerizations. Kim reported a BM method for facilitating the ring‐opening polymerization of d‐lactide in the presence of catalytic amount of an organic base (Scheme 1 C).7d After 2 h of milling, 81 % of the monomer was converted to high MW poly(lactic acid) (PLA). Moreover, the polymer MW correlated with the initial monomer‐to‐initiator ratio ([M]_0_/[I]_0_) and the distributions of polymer chains produced remained relatively low (*Ð*≈1.5). Di‐ and triblock copolymers containing PLA and various hydroxy functionalized macroinitiators, such as poly(ethylene oxide) and poly(*ϵ*‐caprolactone), were subsequently synthesized using similar methodology.7e Kim also reported that the polymerization of trimethylene carbonate in the solid‐state was faster than analogous reactions performed in solution. For example, the polymerization reaction reached 93 % conversion within 2 h when performed in a BM reactor whereas a 70 % conversion was achieved in toluene after 24 h, even though both methods produced polymers of similar MW (9.2 kDa vs. 7.1 kDa, respectively).7f

Although BM may be used to facilitate a range of solid‐state polymerizations, the forces generated during the milling process have been reported to cause chain scission.^9^ For example, the aforementioned poly(phenyl vinylene)s underwent a reduction in MW, from 160 kDa to ca. 40 kDa, within 30 min of BM. Similarly, high MW poly(methyl methacrylate) (255 kDa) became oligomeric (7.6 kDa) after being subjected to BM conditions for 10 h.^10^ The chain scission processes may proceed in a homolytic fashion since radicals have been observed by electron spin resonance spectroscopy upon BM polymeric materials.^11^ It was hypothesized that the radicals generated under such conditions may be harnessed to promote synthetic polymer chemistry. Moreover, if the steady‐state concentration of radicals is sufficiently low, then radical–radical coupling should be suppressed and control over the polymerization reaction may be achieved.

Herein, a variant of atom transfer radical polymerization (ATRP),^12^ which is an efficient reversible‐deactivation radical polymerization method,^13^ was used to facilitate a series of solid‐state BM polymerizations. 2‐Vinylnaphthalene (2‐VN) was selected as the monomer (Scheme 1 D) because it is a solid (mp 64–68 °C) and structurally similar to styrene, a monomer that is commonly polymerized in the solution‐state using ATRP, and thus was envisioned to serve as a model substrate. Initiators and catalysts typically employed in solution based ATRP reactions were used. As will be described below, the polymerizations were found to proceed in a controlled manner as determined by a correlation between the initial monomer‐to‐catalyst ratio ([M]_0_/[I]_0_) and the MW of the polymer produced as well as a series of chain extension experiments. However, the MWs of the polymer products were lower than their theoretical values due to chain scission. To quantify the decomposition processes, models were created to accurately predict polymer MW as a function of milling time. Finally, it will be shown how the technique may be used to prepare copolymers comprised of monomers that exhibit different solubilities and thus be used to circumvent fundamental challenges commonly encountered with the synthesis of such types of materials.

## Results and Discussion

**Figure 1:**
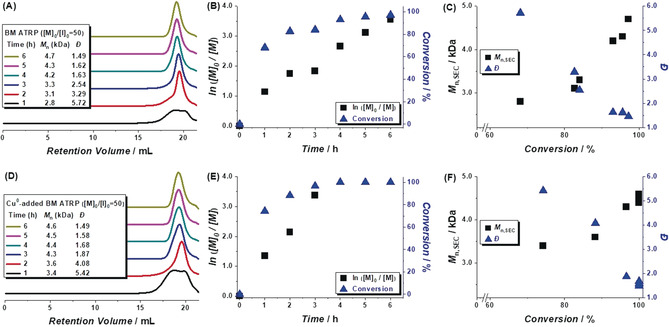
Analyses of BM polymerizations of 2‐vinylnaphthalene (2‐VN) as conducted in the absence (A)–(C) or presence (D)–(F) of a Cu^0^ additive. Conditions: [2‐VN]_0_/[PE‐Br]_0_/[Cu^I^Br/TPMA]_0_=50/1/1 and 20 equiv Cu^0^ for (D)–(F); milling frequency: 30 Hz; N_2_ atmosphere. Polymer molecular weights (*M*_n,SEC_) were measured in THF using SEC and are reported against poly(styrene) standards.

In a preliminary experiment, a zirconium dioxide milling jar was charged with a 50:1:1 molar ratio of 2‐VN, phenylethyl bromide (PE‐Br) (initiator), and Cu^I^Br/tris(2‐pyridylmethyl)amine (TPMA) (catalyst) under nitrogen (N_2_). After adding a 10 mm diameter zirconium dioxide ball and sealing the vessel under N_2_, the mixture was subjected to vibrational BM at 30 Hz for 6 h.^14^ Samples were periodically withdrawn from the vessel and analyzed by size exclusion chromatography (SEC) to monitor the evolution of polymer MW over time or spiked with a standard (anisole) and analyzed by ^1^H NMR spectroscopy to calculate monomer consumption.^15^ As shown in Figure 1 A, the distribution of polymer chains was determined to be relatively broad during the early stages of the reaction, although the polydispersity decreased over time. A semi‐logarithmic plot of the monomer concentration versus time was found to be linear and the conversion of the polymerization reaction reached 97 % after 6 h (Figure 1 B). A linear correlation between the polymer MW and monomer conversion was also observed (Figure 1 C), although the experimentally determined number average MW (*M*_n,SEC_) was lower than its theoretical value (*M*_n,Theory_),^16^ and attributed to premature mechanical degradation (see below). Collectively, these and other results (see Table 1 for a summary) indicated that the solid‐state polymerization reaction was proceeding in a manner consistent with those described for the solution‐state ATRP and other controlled radical polymerization reactions.12b

**Table 1:** Summary of data recorded for BM polymerizations of 2‐vinylnaphthalene.^[a]^

Entry	[M]_0_/[I]_0_/[Cu^I^Br/L]_0_/[Cu^0^]_0_	*t* [h]	Conv. [%]^[b]^	*M*_n,Theory_ [kDa]^[c]^	*M*_n,SEC_ [kDa]^[d]^	*Ð* ^[d]^	Yield [%]^[e]^
1	50/0/0/0	6	*Polymerization was not observed*.				
2^[f]^	50/1/0/0	6					
3	50/1/0/0	6					
4	50/1/1/0	6	97	7.7	4.7	1.46	72
5	50/1/1/20	6	99	7.7	4.6	1.49	85
6	100/1/1/20	4	96	15.0	11.6	1.52	74
7	200/1/1/20	2	84	26.1	21.5	2.09	83
8	300/1/1/20	4	97	45.1	23.9	1.41	83
9	400/1/1/20	3	75	46.4	26.4	1.90	66
10	500/1/1/20	3	94	72.7	28.5	2.68	70

[a] The reactions were conducted in a 10 mL zirconia jar containing a 10 mm diameter ball at 30 Hz under an atmosphere of nitrogen unless otherwise noted. M=2‐VN; I=PE‐Br; L=TMPA. [b] Conversions were determined by ^1^H NMR spectroscopy against anisole as an external standard. [c] The theoretical molecular weights were based on the monomer conversion. [d] Number average molecular weights and molecular weight distributions were obtained by SEC and reported as poly(styrene) equivalents. [e] Isolated yield. [f] AIBN was used as an initiator in *lieu* of PE‐Br.

It has been previously shown that the addition of reductants (e.g., Cu^0^) can accelerate ATRPs without compromising reaction performance or control in part because the additive functions as a supplemental activation and reducing agent.^17^ To determine if such additives would also promote analogous polymerizations in the solid‐state, Cu^0^ powder (20 equiv relative to the initiator) was added to a mixture that was prepared as described above and subjected to the BM conditions. In accord with results obtained in solution,^17^ a faster polymerization reaction was observed (97 % conversion in 3 h) while the relationship between the monomer conversion and the number average MW of the polymer produced remained linear and control over the reaction was achieved (Figures 1 D–F). Considering the advantages bestowed by adding the Cu^0^, subsequent experiments utilized this additive.

To further optimize the BM methodology, the milling frequency was varied. A series of polymerization reactions were independently performed at 10, 20, or 30 Hz for 6 h using a 50:1:1 molar ratio of monomer, initiator, and catalyst in the presence of Cu^0^ (20 equiv). At low frequency, no significant polymerization was observed. However, increasing the frequency to 20 Hz resulted in the formation of a polymer with a *M*_n,SEC_ of 16.0 kDa albeit with a modest monomer conversion (50 %) and relatively broad polydispersity (*Ð* of 3.23). The *M*_n,Theory_, as based on the monomer conversion, was calculated to be lower (4.0 kDa) than the SEC‐derived value, which indicated that the initiation efficiency may be restricted. While the use of a higher milling frequency (30 Hz) resulted in a high monomer conversion (99 %) and afforded a polymer with a relatively low MW (*M*_n,SEC_ of 4.6 kDa) and narrow polydispersity (*Ð* of 1.49), the MW of the polymer produced was found to be lower than its theoretical value (7.8 kDa) and attributed to mechanical degradation during the BM reaction. A series of controls were also performed in parallel with the aforementioned experiments. For example, conducting a polymerization in a ball‐less BM vessel resulted in a monomer conversion of 16 % and afforded a polymer with a *M*_n,SEC_ of 1.0 kDa and *Ð* of 2.27. Likewise, neat polymerizations at 40 °C resulted in a low monomer conversion (38 %) and gave polymers with relatively low MW and high polydispersity index values (*M*_n,SEC_ of 3.3 kDa and *Ð* of 1.69) (see Figure S1).

**Figure 2:**
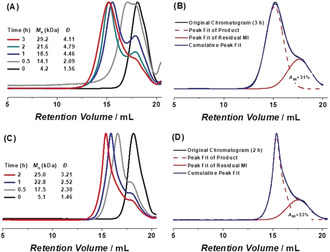
A summary of the evolution of polymer molecular weight during a series of chain extension reactions. The SEC data were recorded over time (indicated) for reactions that utilized a macroinitiator (MI) with a molecular weight of either (A) 4.2 or (C) 5.1 kDa. Conditions: [M]_0_/[MI]_0_/[Cu^I^Br/TPMA]_0_/[Cu^0^]_0_=260/1/1/20; milling frequency: 30 Hz; N_2_ atmosphere. Peak fitting of the size exclusion chromatograms recorded after (B) 3 h for the reaction monitored in (A) or (D) 2 h for the reaction monitored in (C). Legend for (B) and (D): original chromatogram, solid black line (—); peak fit of the signal assigned to the chain extended polymer, dashed red line (‐ ‐ ‐ ‐); peak fit of the residual macroinitiator, solid red line (—); cumulative peak fit, solid blue line (—); and *A*_MI_ for area fraction of MI.

Next, efforts were directed toward verifying that the aforementioned solid‐state polymerizations proceeded in a controlled manner. As summarized in Table 1, a positive correlation between the [M]_0_/[I]_0_ and the polymer MW was observed. While such a relationship reflects a controlled polymerization process, an ability to extend growing polymer chains upon exposure to an additional monomer is a key criterion. To test the latter, low MW macroinitiators were first prepared by BM mixtures containing relatively high loadings of initiator ([M]_0_:[I]_0_:[Cu^I^Br/TPMA]_0_=50:1:1) in the presence of Cu^0^ (20 equiv) for different periods of milling time (3 or 6 h). The *M*_n,SEC_ values of the resulting polymers were measured to be 4.2 and 5.1 kDa, respectively. Each macroinitiator was loaded into a milling jar along with an excess of monomer (260 equiv), Cu^I^Br/TPMA as catalyst (1 equiv), and Cu^0^ (20 equiv), and then subjected to BM (30 Hz). Aliquots were withdrawn from the reaction vessel over time and analyzed using ^1^H NMR spectroscopy and SEC which collectively showed that the monomer was consumed (ca. 80 %) concomitantly with an increase in polymer MW (Figure 2). However, the final products obtained appeared to consist of mainly two distributions of polymer chains: one from the chain extension and one from unreacted macroinitiator. Deconvoluting the corresponding SEC data revealed that the quantity of unreacted macroinitiator was approximately 30 % of the total mixture,^18^ which may be due to a loss of the halogen end‐groups during the macroinitiator synthesis or chain extension.

To quantify the apparent loss in end‐group functionality over time, a low MW polymer was synthesized using the BM methodology described above ([M]_0_:[I]_0_:[Cu^I^Br/TPMA]_0_:[Cu^0^]_0_=50:1:1:20). After 2 h, 53 % of the monomer was converted to polymer, as determined by analyzing the product mixture using ^1^H NMR spectroscopy. Based on the monomer conversion value and assuming full initiation, the *M*_n,Theory_ of the polymer produced was calculated to be 4.3 kDa. Further inspection of the NMR data revealed diagnostic signals at δ 4.5 ppm and over the range of 2.7 to 0.5 ppm (CDCl_3_), which were assigned to the terminal bromomethine groups and hydrogens in the polymer backbone, respectively. Using the relative intensities of the aforementioned NMR signals, the number average MW of the polymer (*M*_n,NMR_) was calculated to be 6.7 kDa (see Figure S2). The difference between the *M*_n,Theory_ and *M*_n,NMR_ values indicated that approximately 33 % of the chain termini became non‐functional during the polymerization reaction. For comparison, approximately 8 % of the end‐groups lose their functionality during the solution phase ATRP of styrene at similar conversions (48 %).^19^

A hallmark of solution‐state ATRP reactions is that they proceed through radical pathways as determined in part through trapping experiments with scavengers (e.g., 2,2,6,6‐tetramethyl‐1‐piperidinyloxy (TEMPO) free radical).^20^ To determine if radicals were also generated during the aforementioned solid‐state ATRP and germane to the process, a series of reactions were conducted in the presence of TEMPO. A mixture of the monomer, initiator, catalyst, reductant, and TEMPO ([M]_0_:[I]_0_:[Cu^I^Br/TPMA]_0_:[Cu^0^]:[TEMPO]_0_=50:1:1:20:2) was subjected to BM conditions and monitored by ^1^H NMR spectroscopy as well as SEC over time (see Figure S3). After 6 h, less than 30 % of the monomer was consumed and a low yield of an oligomer (*M*_n,SEC_=0.7 kDa) was obtained. Moreover, signals consistent with a TEMPO adduct (C*H_3_*‐, δ 1.47 ppm in CDCl_3_) were observed upon ^1^H NMR analysis of the product. Likewise, ball milling a mixture of initiator, catalyst, reductant, and TEMPO ([I]_0_:[Cu^I^Br/TPMA]_0_:[Cu^0^]_0_:[TEMPO]_0_=1:1:20:2) generated phenylethyl TEMPO (PE‐TEMPO) in 92 % yield within 30 min, as determined by ^1^H NMR spectroscopy. For comparison, no reaction was observed when an analogous reaction was performed without catalyst, even after extended periods of time (see Figure S4). Similarly, no polymerization was observed when only the monomer or the monomer and a typical free radical initiator (e.g., azobisisobutyronitrile; AIBN) were separately subjected to the BM conditions (see Table 1). Collectively, these results indicated that the solid‐state ATRP reactions initiated rapidly and proceeded in a manner similar to those that are performed in solution, and that the catalyst was key to not only generating radicals but also maintaining their concentrations at a steady state.

**Figure 3:**
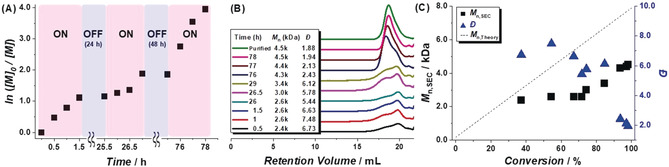
Summary of polymerization kinetics data that were recorded over time. (A) A semi‐logarithmic plot of the monomer concentration vs. time. Note that the areas labeled as “on” or “off” state refer to periods wherein the BM frequency was varied between 30 Hz and 0 Hz, respectively. (B) Size exclusion chromatograms and corresponding data as recorded over time (indicated). Note: the chromatogram labeled as “Purified” refers to data that were recorded for a polymer that was passed through a column of neutral alumina and then precipitated from methanol. (C) Plot of experimentally determined polymer MW (*M*_n,SEC_) and polydispersity index values (*Ð*) vs. the percentage of monomer that converted to polymer. Conditions: [2‐VN]_0_/[PE‐Br]_0_/[Cu^I^Br/TPMA]_0_/[Cu^0^]_0_=50/1/1/20; Milling frequency: 30 Hz; N_2_ atmosphere. Aliquots were periodically withdrawn from the reaction vessel, spiked with a known quantity of a standard (anisole), and then analyzed by ^1^H NMR spectroscopy (CDCl_3_) to determine the conversion. The *M*_n,SEC_ values are reported as their poly(styrene) equivalents.

Since various stimuli (e.g., photochemical,^21^ electrochemical,^22^ and sonochemical^23^) have been used to effectively switch ATRP reactions between “on” (active) and “off” (inactive) states over time, it was reasoned that intermittently varying the BM frequency over the course of a polymerization reaction should also enable temporal control. A mixture of monomer, initiator, catalyst, and reductant ([M]_0_:[I]_0_:[Cu^I^Br/TPMA]_0_:[Cu^0^]_0_=50:1:1:20) was subjected to BM conditions at 30 Hz for different periods of time. As shown in Figure 3, the rate of the polymerization was multiply switched between “on” and “off” states over the course of 78 h by alternating the milling frequency. While chain growth occurred only during the “on” states, the resulting the polymer exhibited a relatively broad polydispersity (*Ð* of 1.88), presumably due to the chain‐end deactivation processes described above and/or mechanical degradation.

As noted above, the MWs of the polymers produced were measured to be lower than their theoretical values and attributed to chain scission (e.g., see Figure S5 for plots of key data obtained from Table 1). As such, the phenomenon was modeled to gain a deeper understanding of the decomposition mechanism and to predict the loss in polymer MW. As summarized in Equation (1), the decomposition rate can be expressed in terms of the change in polymer MW over time and the corresponding rate constant (*k*_d_) can thus be determined from the semi‐logarithmic relationship described in Equation (2). An integrated form of the latter, Equation (3), indicates that the polymer MW at any given time (*M*_n,t_) should exponentially decrease from its initial state (*M*_n,0_) and approach a limiting value (*M*_n,∞_).^9^, ^11^ Assuming that a polymerization reaction affords a polymer with its theoretical MW (*M*_n,Theory_) if there was no decomposition, the *M*_n,0_ can be equated to *M*_n,Theory_ and thus the loss in polymer MW (*M*_n,Loss_) can be determined as a function of milling time, as shown in Equation (4).

(1) $$R_{d}=-dM_{n}\;/\;\;dt=k_{d}\left(M_{n}-M_{n,\infty }\right)$$

(2) $$\ln\left[\frac{M_{n,0}-M_{n,\infty }}{M_{n,t}-M_{n,\infty }}\right]=k_{d}t$$

(3) $$M_{n,t}=\left(M_{n,0}-M_{n,\infty }\right)e^{-k_{d}t}+M_{n,\infty }$$

(4) $$M_{n,\mathrm{Loss}}=\left(M_{n,\mathrm{Theroy}}-M_{n,\infty }\right)e^{-k_{d}t}+M_{n,\infty }$$

where *t*, *R*_d_, *M*_n,0_, *M*_n,*t*_, *M*_n,Loss_, and *M*_n,Theory_ are the milling time, the rate of decomposition, the initial polymer molecular weight, the polymer molecular weight at time *t*, the predicted loss in polymer molecular weight due to mechanical degradation, and the theoretical molecular weight, respectively.

**Figure 4:**
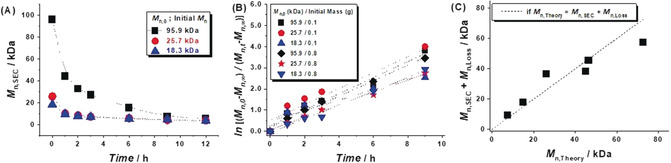
Modeling of polymer molecular weight (MW) vs. time. (A) MW decrement as determined by SEC using polymers with three different initial MWs (*M*_n,0_): 95.9 (black ▪), 25.7 (red •), and 18.3 kDa (blue ▴). (B) Semi‐logarithmic plot of MW decrement vs. milling time [see Eq. (2)]. (C) Plot of the experimentally determined MW (*M*_n,SEC_) plus the modeled loss in polymer MW (*M*_n,Loss_) vs. the theoretical MW (*M*_n,Theory_).

To test the aforementioned model, a series of decomposition studies were conducted by separately BM poly(2‐VN) with different initial MWs (*M*_n,0_=95.9, 25.7, or 18.3 kDa). After 12 h, the MWs of the polymers measured for each experiment approached a limiting *M*_n,∞_ value of 3.2 kDa (Figure 4 A).^9^ The *k*_d_ values measured from the semi‐logarithmic plots of the change in MW versus milling time were found to be similar and an average of 0.33±0.054 h^−1^ was calculated (Figure 4 B). Inputting the *k*_d_ value into Equation (4) resulted in a linear correlation between *M*_n,Theory_ and the experimentally determined MW (*M*_n,SEC_) summed with the predicted loss in polymer molecular weight (*M*_n,Loss_) (Figure 4 C). The good fit indicates that the model not only effectively rationalizes the difference between *M*_n,Theory_ and *M*_n,SEC_ but provides a means to predict polymer MW as a function of BM time.

To realize the potential of the aforementioned methodology, efforts were directed toward the synthesis of random copolymers comprised of charged and neutral monomers. Such copolymers, which are often termed polyelectrolytes,^24^ have found utility in applications that range from nanoparticle encapsulation^25^ to drug delivery,^26^ yet are challenging to prepare because the two types of monomers typically exhibit orthogonal solubilities.^27^ As a result, relatively sophisticated synthetic schemes that often entail multiple protection–deprotection steps are required,^28^ even when controlled radical polymerizations are used.^29^ The solid‐state BM ATRP method described above employs a single phase and thus effectively circumvents these fundamental and practical drawbacks. To maintain continuity with the aforementioned studies, 2‐VN was selected as a monomer along with sodium styrene sulfonate (NaSS), a charged species that is often paired with neutral monomers in the synthesis of copolymers.^30^ As summarized in Table 2, various mixtures of 2‐VN and NaSS were combined with the initiator, catalyst, and reductant described above, and then ball milled at 30 Hz. Aliquots were periodically withdrawn from the reaction vessel and dissolved in either CDCl_3_ (for 2‐VN) or D_2_O (for NaSS), spiked with a known quantity of an external standard (anisole or DMF, respectively) and analyzed by ^1^H NMR spectroscopy to ascertain monomer conversion (see Figure S6). As expected, the solubilities of the copolymer products depended on their compositions. Copolymers with relatively high molar compositions of 2‐VN (e.g., poly(2‐VN)_28_‐*ran*‐poly(NaSS)_7_) were soluble in organic solvents whereas copolymers rich in NaSS (e.g., poly(2‐VN)_10_‐*ran*‐poly(NaSS)_36_) were soluble in aqueous media. Copolymers with near equimolar monomer compositions (e.g., poly(2‐VN)_25_‐*ran*‐poly(NaSS)_18_) were insoluble in THF as well as aqueous media and could only be dissolved in DMSO at elevated temperatures. The solubility differential required the development of a novel suite of techniques to characterize the copolymers. SEC was used to determine the *M*_n_ and the polydispersity of the copolymers that were soluble in either THF or aqueous media. However, to facilitate a universal comparison, dynamic light scattering (DLS) was used in conjunction with the specific refractive index increment (dn/dc) of the copolymers, which was found to be linearly correlated with the monomer composition in DMSO (see Figure S7) and was used to determine the absolute weight average MWs (*M*_w,absol._) of the copolymers. Collectively, the MWs and polydispersities of the copolymers were typical of controlled polymerizations and, in a broader perspective, the results demonstrated that the solid‐state methodology may facilitate access to copolymers that are inaccessible or challenging to prepare via solution‐state approaches.^31^

**Table 2:** Summary of data recorded for BM polymerizations of 2‐vinylnaphthalene (2‐VN) and sodium styrene sulfonate (NaSS).^[a]^

Entry	Label	Conv. [%] of 2‐VN/NaSS^[b]^	*M*_n,Theory_ [kDa]^[c]^	*M*_n,SEC_ [kDa]^[d]^	*Ð* ^[d]^	dn/dc^[e]^	*M*_w,absol._ [kDa]^[f]^	Yield [%]^[g]^
1	poly(2‐VN)_50_	99/–	7.9	4.8^[h]^	1.53	0.1604	19.1	85
2	poly(2‐VN)_28_‐*ran*‐poly(NaSS)_7_	70/71	6.0	4.8^[h]^	2.29	0.1380	22.2	82
3	poly(2‐VN)_25_‐*ran*‐poly(NaSS)_18_	99/72	7.8	n.d.	n.d.	0.1120	25.2	91
4	poly(2‐VN)_10_‐*ran*‐poly(NaSS)_36_	99/91	9.2	18.0^[i]^	1.22	0.0934	20.5	88
5	poly(NaSS)_50_	–/99	10.5	12.5^[i]^	1.10	0.0807	16.1	89

[a] The reactions were conducted in a 10 mL zirconia jar containing a 10 mm diameter ball at 30 Hz under an atmosphere of nitrogen for 4 h unless otherwise noted. [b] Conversions were determined by ^1^H NMR spectroscopy against an external standard: either anisole for 2‐VN in CDCl_3_ or DMF for NaSS in D_2_O. [c] The theoretical molecular weights were based on the monomer conversion. [d] Number average molecular weights and molecular weight distributions were obtained by SEC. [e] The specific refractive index increments were measured in DMSO at 50 °C. [f] Weight average molecular weights were measured via dynamic light scattering at 663 nm in DMSO at 50 °C. [g] Isolated yield. [h] Determined in THF against poly(styrene) standards. [i] Determined in aqueous media (0.2 m NaNO_3_ and 0.01 m NaH_2_PO_4_ with 30 % methanol at pH 7) against poly(ethylene oxide) standards. n.d.=not determined.

## Conclusion

In conclusion, a series of ATRP reactions were performed in the solid‐state. BM mixtures that consisted of initiators and catalysts commonly employed in solution‐state ATRP reactions along with solid monomers resulted in controlled polymerizations, and the addition of Cu^0^ accelerated the reactions without detriment. Radicals were generated during the process, as confirmed by trapping experiments, and appeared to reach a steady state within a short period time. Moreover, the polymerization reaction was effectively switched between active and inactive states by alternating the applied frequency over time. While losses in end‐group functionality were observed and the molecular weights of the polymers produced were lower than their theoretical values, the differences, which were attributed to mechanically induced chain scission, were successfully modeled and an accurate prediction of the polymer MW over time was realized. In a broader context, these results demonstrate that radicals generated in the solid‐state may be harnessed in a similar manner to those formed in solution. Moreover, copolymers that are inaccessible or challenging to obtain via solution‐state polymerization methods were also synthesized. As such, the solid‐state chemistry described herein may effectively obviate the need for solvents in other types of radical‐based, synthetic transformations (e.g., Kharasch additions, reversible addition‐fragmentation chain‐transfer (RAFT), etc.) and expedite access to exotic polymeric materials that exhibit limited solubilities in organic solvents or aqueous media.

## Conflict of interest

The authors declare no conflict of interest.

## Supporting information

As a service to our authors and readers, this journal provides supporting information supplied by the authors. Such materials are peer reviewed and may be re‐organized for online delivery, but are not copy‐edited or typeset. Technical support issues arising from supporting information (other than missing files) should be addressed to the authors.

Supplementary

## References

[1] S. L. James , C. J. Adams , C. Bolm , D. Braga , P. Collier , T. Friscic , F. Grepioni , K. D. M. Harris , G. Hyett , W. Jones , A. Krebs , J. Mack , L. Maini , A. G. Orpen , I. P. Parkin , W. C. Shearouse , J. W. Steed , D. C. Waddell , Chem. Soc. Rev. 2012, 41, 413–447. 21892512 10.1039/c1cs15171a

[2] A. Stolle , T. Szuppa , S. E. S. Leonhardt , B. Ondruschka , Chem. Soc. Rev. 2011, 40, 2317–2329. 21387034 10.1039/c0cs00195c

[3a] F. T. Edelmann , Coord. Chem. Rev. 2016, 306, 346–419;

[3b] K. Kubota , Y. Pang , A. Miura , H. Ito , Science 2019, 366, 1500–1504. 31857482 10.1126/science.aay8224

[4] L. Peltonen , J. Hirvonen , J. Pharm. Pharmacol. 2010, 62, 1569–1579. 21039542 10.1111/j.2042-7158.2010.01022.x

[5] X. Y. Guo , D. Xiang , G. H. Duan , P. Mou , Waste Manage. 2010, 30, 4–10. 10.1016/j.wasman.2009.08.017 19811900

[6] H. Watanabe , E. Matsui , Y. Ishiyama , M. Senna , Tetrahedron Lett. 2007, 48, 8132–8137.

[7a] J. B. Ravnsbæk , T. M. Swager , ACS Macro Lett. 2014, 3, 305–309; 10.1021/mz500098r 35590736

[7b] S. Grätz , L. Borchardt , RSC Adv. 2016, 6, 64799–64802;

[7c] S. Gratz , B. Wolfrum , L. Borchardt , Green Chem. 2017, 19, 2973–2979;

[7d] N. Ohn , J. Shin , S. S. Kim , J. G. Kim , ChemSusChem 2017, 10, 3529–3533; 28613397 10.1002/cssc.201700873

[7e] G. S. Lee , B. R. Moon , H. Jeong , J. Shin , J. G. Kim , Polym. Chem. 2019, 10, 539–545;

[7f] S. Park , J. G. Kim , Beilstein J. Org. Chem. 2019, 15, 963–970; 31164933 10.3762/bjoc.15.93 PMC6541340

[7g] Z. Wang , J. Ayarza , A. P. Esser-Kahn , Angew. Chem. Int. Ed. 2019, 58, 12023–12026; 10.1002/anie.201903956 31267620

[7h] J. Collins , T. G. McKenzie , M. D. Nothling , S. Allison-Logan , M. Ashokkumar , G. G. Qiao , Macromolecules 2019, 52, 185–195.

[8a] J. L. Do , C. Mottillo , D. Tan , V. Strukil , T. Friscic , J. Am. Chem. Soc. 2015, 137, 2476–2479; 25668586 10.1021/jacs.5b00151

[8b] R. Thorwirth , A. Stolle , B. Ondruschka , A. Wild , U. S. Schubert , Chem. Commun. 2011, 47, 4370–4372; 10.1039/c0cc05657j 21399799

[8c] D. A. Fulmer , W. C. Shearouse , S. T. Medonza , J. Mack , Green Chem. 2009, 11, 1821–1825.

[9] J. Sohma , Prog. Polym. Sci. 1989, 14, 451–596.

[10] A. P. Smith , J. S. Shay , R. J. Spontak , C. M. Balik , H. Ade , S. D. Smith , C. C. Koch , Polymer 2000, 41, 6271–6283.

[11] S. I. Kondo , Y. Sasai , S. Hosaka , T. Ishikawa , M. Kuzuya , J. Polym. Sci. Part A 2004, 42, 4161–4167.

[12a] K. Matyjaszewski , N. V. Tsarevsky , Nat. Chem. 2009, 1, 276–288; 21378870 10.1038/nchem.257

[12b] J. F. Lutz , J. M. Lehn , E. W. Meijer , K. Matyjaszewski , Nat. Rev. Mater. 2016, 1, 14.

[13a] G. Moad , E. Rizzardo , S. H. Thang , Acc. Chem. Res. 2008, 41, 1133–1142; 18700787 10.1021/ar800075n

[13b] W. A. Braunecker , K. Matyjaszewski , Prog. Polym. Sci. 2007, 32, 93–146.

[14] Polymer was not observed when an analogous reaction was set up and conducted under an atmosphere of air.

[15] Using an IR thermometer, the temperature of the reaction vessel was measured to range from 35 °C (outside) to 40 °C (inside) after being subjected to the BM conditions for 6 h.

[16] *M* _n,Theory_=MW of initiator + (MW of monomer × monomer conversion × [M]_0_/[I]_0_).

[17] K. Matyjaszewski , S. Coca , S. G. Gaynor , M. L. Wei , B. E. Woodworth , Macromolecules 1997, 30, 7348–7350.

[18] Peak fitting was accomplished using the Gaussian function as implemented in the OriginPro 8 software package.

[19] J.-F. Lutz , K. Matyjaszewski , J. Polym. Sci. Part A 2005, 43, 897–910.

[20] K. Matyjaszewski , B. Göbelt , H.-j. Paik , C. P. Horwitz , Macromolecules 2001, 34, 430–440.

[21] D. Konkolewicz , K. Schroder , J. Buback , S. Bernhard , K. Matyjaszewski , ACS Macro Lett. 2012, 1, 1219–1223. 10.1021/mz300457e 35607200

[22] A. J. Magenau , N. C. Strandwitz , A. Gennaro , K. Matyjaszewski , Science 2011, 332, 81–84. 21454784 10.1126/science.1202357

[23a] H. Mohapatra , M. Kleiman , A. P. Esser-Kahn , Nat. Chem. 2017, 9, 135–139;

[23b] Z. Wang , F. Lorandi , M. Fantin , Z. Wang , J. Yan , Z. Wang , H. Xia , K. Matyjaszewski , ACS Macro Lett. 2019, 8, 161–165. 10.1021/acsmacrolett.9b00029 35619423

[24] R. R. Netz , D. Andelman , Phys. Rep. 2003, 380, 1–95.

[25] J.-F. Berret , N. Schonbeck , F. Gazeau , D. El Kharrat , O. Sandre , A. Vacher , M. Airiau , J. Am. Chem. Soc. 2006, 128, 1755–1761. 16448152 10.1021/ja0562999

[26] M. Elsabahy , K. L. Wooley , Chem. Soc. Rev. 2012, 41, 2545–2561. 22334259 10.1039/c2cs15327k PMC3299918

[27] P. Raffa , D. A. Z. Wever , F. Picchioni , A. A. Broekhuis , Chem. Rev. 2015, 115, 8504–8563. 26182291 10.1021/cr500129h

[28a] Q. Zhang , E. E. Remsen , K. L. Wooley , J. Am. Chem. Soc. 2000, 122, 3642–3651;

[28b] H. Okamura , Y. Takatori , M. Tsunooka , M. Shirai , Polymer 2002, 43, 3155–3162.

[29] R. K. O'Reilly , M. J. Joralemon , C. J. Hawker , K. L. Wooley , J. Polym. Sci. Part A 2006, 44, 5203–5217.

[30] W. Liu , A. L. Cholli , R. Nagarajan , J. Kumar , S. Tripathy , F. F. Bruno , L. Samuelson , J. Am. Chem. Soc. 1999, 121, 11345–11355.

[31] M. Nowakowska , S. Zapotoczny , A. Karewicz , Macromolecules 2000, 33, 7345–7348.

