# Supplementary material for: Atom Transfer Radical Polymerization in the Solid‐State
Source: Angew Chem Int Ed Engl. 2020 Jun 8;59(33):13929–35. doi: 10.1002/anie.202005021 (PMC7496184; doi:10.1002/anie.202005021)
Supplement: Supplementary file 1 — Supplementary [file ANIE-59-13929-s001.pdf]

Angewandte Chemie
Eine Zeitschrift der Gesellschaft Deutscher Chemiker

Supporting Information

Atom Transfer Radical Polymerization in the Solid-State

*Hong Y. Cho and Christopher W. Bielawski**

anie_202005021_sm_miscellaneous_information.pdf

Author Contributions

All authors have given approval to the final version of the manuscript.

Characterization

Size exclusion chromatography (SEC) was performed on a Malvern Viscotek GPCmax system. Two fluorinated polystyrene columns (IMBMW-3078) were used in series and maintained at 35 °C. THF was used as the mobile phase at a flow rate of 0.8 mL/min. Detection was performed using a Malvern Viscotek Triple Detector Array (TDA305) system. The chromatograph was calibrated against poly(styrene) standards. Aqueous SEC was performed on an Agilent 1260 system at 35 °C and equipped with a PL aquagel-OH (8 µm) guard as well as two PL aquagel-OH Mixed-M (8 µm) columns. An aqueous solution (pH 7) that contained $NaNO_3$ (0.2 M), NaH_2PO_4 (0.01 M) and methanol (30%) was used as the eluent. The aqueous chromatograph was calibrated against poly(ethylene oxide) standards. Refractive index values were measured using an Anton Paar Abbemat 200 refractometer. Dynamic light scattering was performed on a DynaPro NanoStar at 663 nm. ^{1}H NMR spectra were recorded using a Bruker 400 MHz spectrometer and analyzed using the MestReNova software package. Matrix-assisted laser desorption and ionization time-of-flight mass spectrometry (MALDI-TOF) measurements were carried out on an Ultraflex III MALDI mass spectrometer with a dithranol matrix.

Materials

2-Vinylnaphthalene (2-VN; Alfa Aesar, 97%) was purified by passing through basic alumina in THF followed by sublimation. Cu^IBr (Sigma-Aldrich, 98%) and Cu^0 powder (Sigma-Aldrich, 98%) were purified in glacial acetic acid by stirring for 12 hours followed by washing with ethanol before being dried under reduced pressure. Azobisisobutyronitrile (AIBN; Junsei Chemical, 98%) was recrystallized from cold methanol prior to use. 1-Phenylethyl bromide (PE-Br; Sigma-Aldrich, 97%), tris(2-pridylmethyl)amine (TPMA; Sigma-Aldrich, 98%), 2,2,6,6-tetramethyl-1-piperidinyloxy (TEMPO; Sigma-Aldrich, 98%), sodium 4-styrene sulfonate (NaSS; Sigma-Aldrich, ≥90%), poly(2-vinylnaphthalene) (poly(2-VN); Sigma-Aldrich, M_n of 95.9 kDa and Scientific Polymer Products, M_n of 18.3 kDa), and all solvents were used without purification.

General Synthetic Considerations

Ball milling atom transfer radical polymerizations were typically conducted with an initiator (PE-Br), monomer (2-VN), and catalyst (Cu^IBr / TPMA) in the presence of reductant (Cu^0). Reagent ratios are described elsewhere. The initiator, monomer, catalyst, and reductant were added in the order listed into a 10 mL zirconium oxide milling jar equipped with a zirconium oxide ball (10 mm diameter), followed by purging with nitrogen gas. The jar was subjected to Retsch mixer mill 400 (MM400) at 30 Hz that was placed in a nitrogen-charged glove-bag. Aliquots were withdrawn from the reaction vessel under nitrogen over time and then analyzed as described in the main text. Monomer conversions were determined using ^{1}H NMR spectroscopy with anisole (or DMF) as an external standard in $CDCl_3$ (or D_2O). Size exclusion chromatography (SEC) was used to determine the number average molecular weight ($M_{n,SEC}$) and molecular weight distribution ($Đ$) data. Poly(2-VN) with a M_n of 25.7 kDa and a $Đ$ of 1.59 was synthesized by reversible addition-fragmentation transfer polymerization using 2-cyano-2-propyl dodecyl trithiocarbonate, 2-VN, and 1,1′-azobis(cyclohexanecarbonitrile) (1 : 500 : 0.5, respectively) in 50% DMF at 100 °C for 18 h (35% yield).

WILEY-VCH

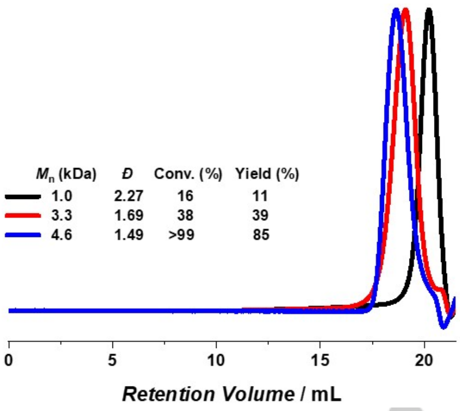

Figure S1. Comparison of GPC data recorded for poly(2-VN) as prepared in the solid-state under different conditions: neat polymerization at 40 °C (—), milling without a ball (—), and BM with a ball (—). Conditions: $[2\text{-VN}]_0$ / $[\text{PE-Br}]_0$ / $[\text{Cu}^{I}\text{Br / TPMA}]_0$ / $[\text{Cu}^0]_0$ = 50 / 1 / 1 / 20; milling frequency: 30 Hz; N_2 atmosphere; 6 h.

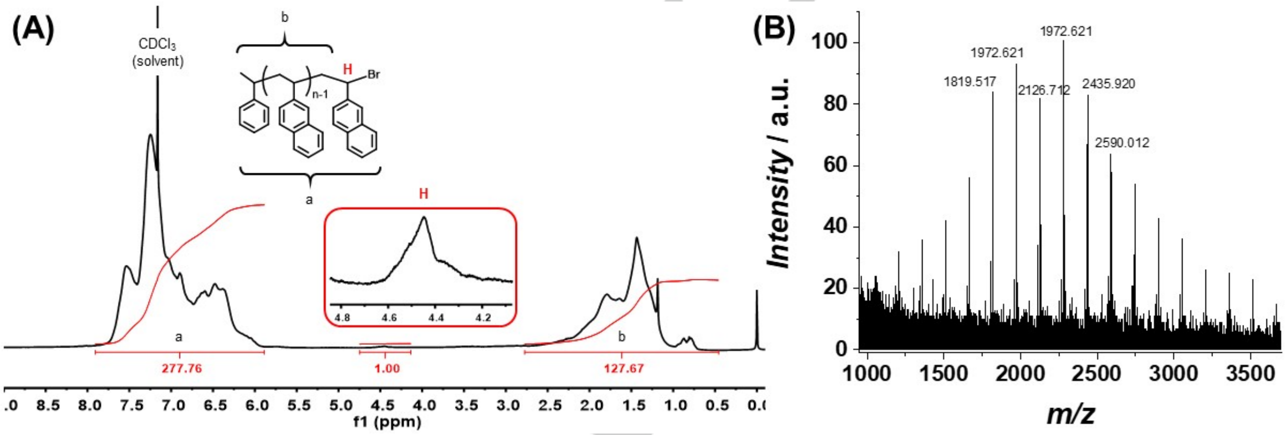

Figure S2. Additional characterization data that were obtained for poly(2-VN). Polymerization conditions: $[2\text{-VN}]_0$ / $[\text{PE-Br}]_0$ / $[\text{Cu}^{I}\text{Br / TPMA}]_0$ / $[\text{Cu}^0]_0$ = 50 / 1 / 1 / 20; milling frequency: 30 Hz; N_2 atmosphere; 2 h. (A) ^{1}H NMR (400 MHz) spectrum as recorded in $CDCl_3$ at 25 °C (the $M_{n,\text{NMR}}$ was determined to be 6.7 kDa) and (B) MALDI-TOF mass data recorded for the polymer using a dithranol matrix. The difference in mass signals was determined to be 154.099 and consistent with a polymer containing 2-VN (*m/z* = 154.08) as the repeating unit.

WILEY-VCH

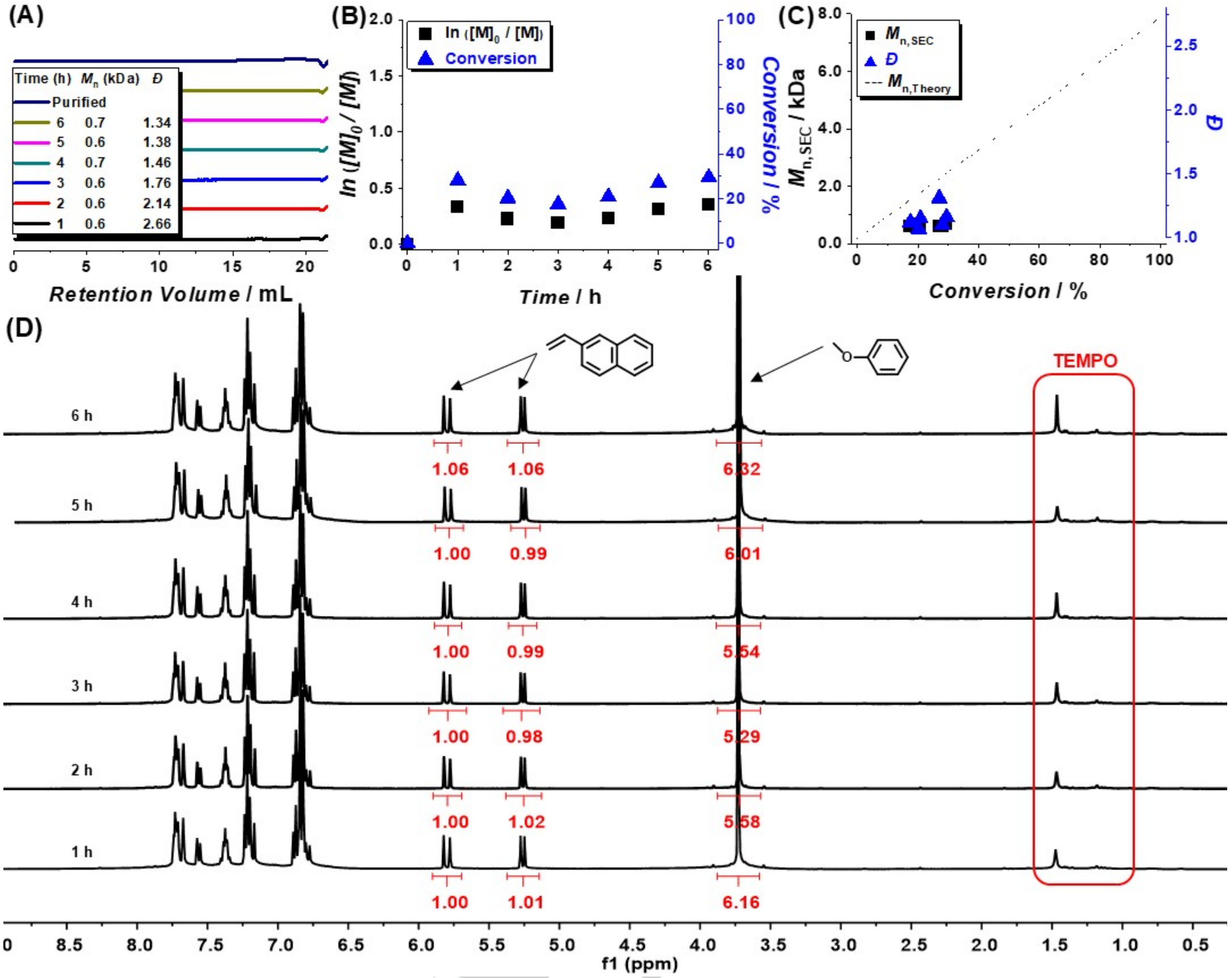

Figure S3. Summary of data recorded for a series of polymerization reactions that were conducted in the presence of TEMPO. Conditions: $[2\text{-VN}]_0$ / $[\text{PE-Br}]_0$ / $[\text{Cu}^{\text{I}}\text{Br} / \text{TPMA}]_0$ / $[\text{Cu}^0]_0$ / $[\text{TEMPO}]_0$ = 50 / 1 / 1 / 20 / 2; milling frequency: 30 Hz; N_2 atmosphere. (A) SEC data as recorded over time (indicated). Note: the chromatogram labeled as "Purified" refers to data recorded for a polymer that was passed through a neutral alumina column followed by precipitation from methanol. (B) Semi-logarithmic plot of the monomer concentration and monomer conversion versus time. (C) SEC data as a function of monomer conversion. (D) ^{1}H NMR spectra recorded over time (indicated) with an added standard (anisole).

WILEY

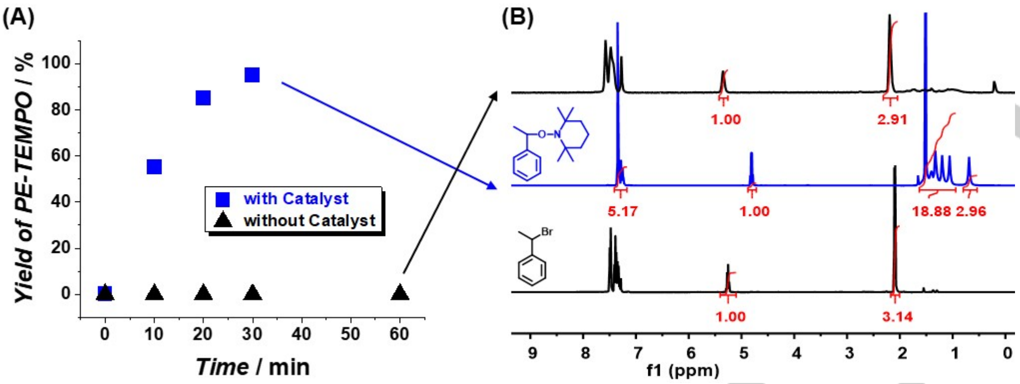

Figure S4. Summary of radical trapping experiments. Conditions: $[PE\text{-}Br]_0$ / $[Cu^{I}Br / TPMA]_0$ / $[Cu^0]_0$ / $[TEMPO]_0$ = 1 / 1 / 20 / 2; milling frequency: 30 Hz; N_2 atmosphere. (A) Plot of yield of PE-TEMPO versus milling time in the presence of catalyst (■) or absence of catalyst (▲). The PE-TEMPO product was purified by column chromatography (silica gel; eluent = hexane : dichloromethane = 9 : 1 v/v). (B) ^{1}H NMR spectra recorded for PE-Br (bottom) and the indicated samples in $CDCl_3$ at 25 °C.

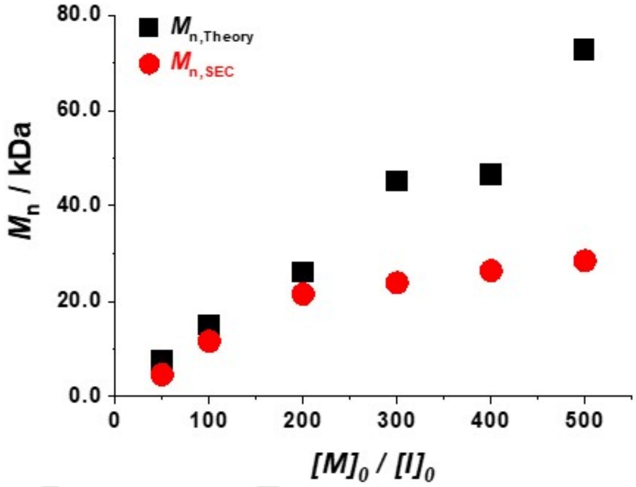

Figure S5. Plots of polymer molecular weight versus the initial monomer-to-initiator ratio ($[M]_0$ / $[I]_0$). Data were taken from Table 1. The theoretical molecular weight values ($M_{n,Theory}$, ■) were calculated as follows: MW of initiator + (MW of monomer × monomer conversion × $[M]_0$ / $[I]_0$). The experimentally determined molecular weight values ($M_{n,SEC}$, ●) were obtained using SEC. Polymerization conditions: $[M]_0$ / $[I]_0$ / $[Cu^{I}Br / TPMA]_0$ / $[Cu^0]_0$ = 50 to 500 / 1 / 1 / 20; milling frequency: 30 Hz; N_2 atmosphere.

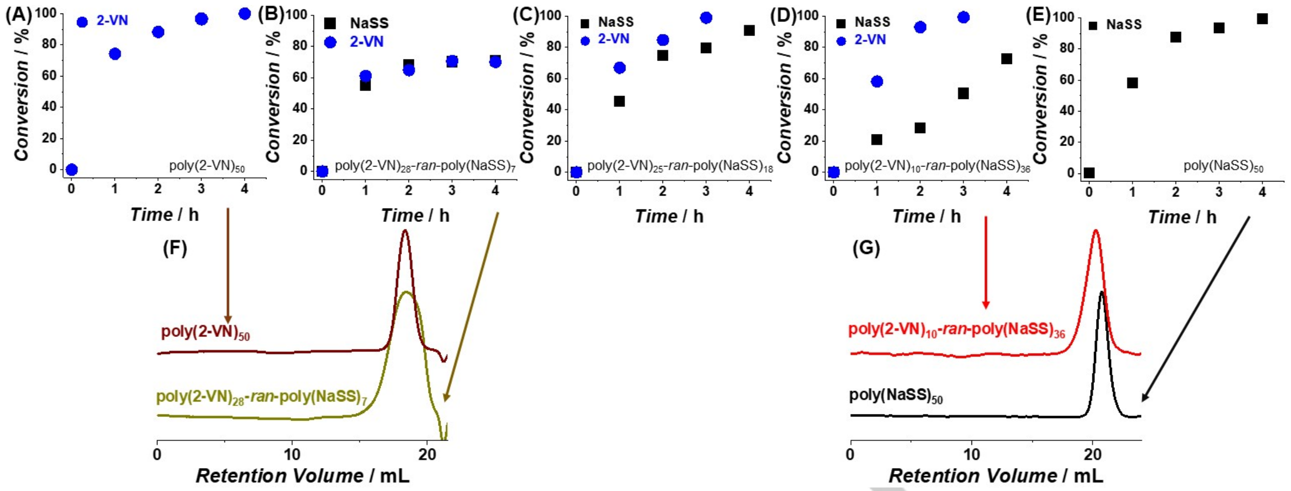

Figure S6. Plots of monomer conversion and SEC data recorded during BM (co)polymerizations of 2-VN and/or NaSS. (A-E) Conversions of 2-VN (●) and NaSS (■) to polymer over time (indicated). The monomer conversions were determined by periodically extracting aliquots from the reaction mixtures, diluting the aliquots with $CDCl_3$ for 2-VN or D_2O for NaSS, and finally analyzing the diluted aliquots using ^1H NMR spectroscopy after spiking with known quantities of anisole or DMF, respectively. (F) The SEC data for poly(2-VN)$_{50}$ and poly(2-VN)$_{28}$-*ran*-poly(NaSS)$_7$ were recorded in THF. (G) The SEC data for poly(NaSS)$_{50}$ and poly(2-VN)$_{10}$-*ran*-poly(NaSS)$_{36}$ were recorded in aqueous media.

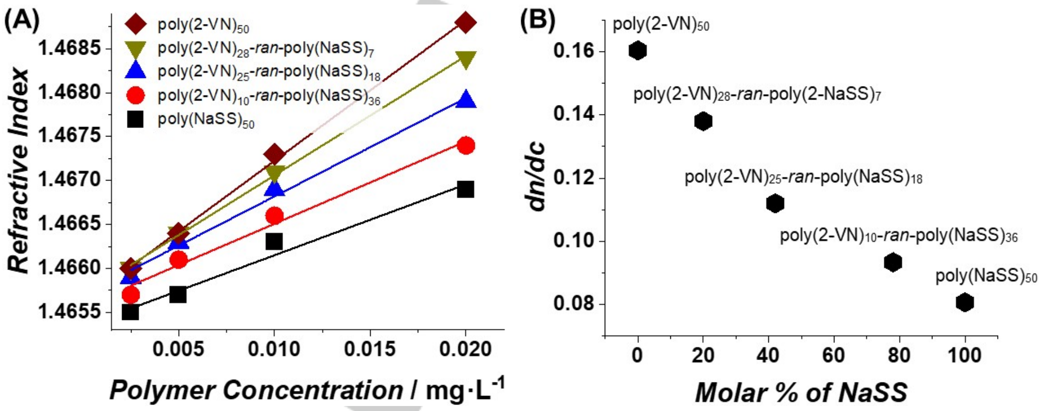

Figure S7. Summary of polymer optical properties. (A) Plots of refractive index value versus concentration of various polymers (indicated) as measured in DMSO at 50 °C. (B) Plot of dn/dc versus the molar percentage of NaSS in various polymers (indicated).
